# Sand dam contributions to year-round water security monitored through telemetered handpump data

**DOI:** 10.1007/s10661-023-11694-9

**Published:** 2023-10-17

**Authors:** Hannah Ritchie, Ian Holman, Alison Parker, Joanna Chan

**Affiliations:** Cranfield Centre for Water, Environment and Development, Cranfield University, Bedford, UK

**Keywords:** Dryland, Rainwater harvesting, Telemetry, Arid-semi-arid land (ASAL), Abstraction, Kenya

## Abstract

Sand dams are a form of rainwater harvesting, prolific in arid and semi-arid lands. Water is provided partly via handpumps, which, as the only improved method of abstraction from sand dams, are important for drinking water security. Accelerometers and cellular transmitters were fitted to 30 handpumps by the Africa Sand Dam Foundation (ASDF) in 2019 to monitor the use and reliability of the handpumps by recording hourly water volume abstracted. Data from April 2019 to October 2021 for 26 of these sites, alongside qualitative data, were analysed and each handpump’s contribution to year-round water security was explored, focusing on the long dry season when water supply from other sources is compromised. Abstraction was over 20 times higher in the long dry season than in any other season, and at sites with higher salinity, higher livestock use, and larger dam wall area. At 21 wells, abstraction was still being recorded at the end of at least one long dry season; however, high spatial and temporal heterogeneity between pumps and seasons means that not all sand dams deliver reliable water supply year-round. Quantifying the contribution that sand dams make to water security is crucial for understanding their resilience against a changing climate and can aid decision makers when choosing the most appropriate water management technique. Knowledge of temporal and site heterogeneity in abstraction can inform when other water sources need increasing and can help with sand dam design optimisation. Overall, our results indicate the positive contribution that sand dams make to year-round water security through the water that is abstracted through handpumps.

**Supplementary Information:**

The online version contains supplementary material available at 10.1007/s10661-023-11694-9.

## Introduction

Drylands, or arid regions, are home to one-third of the world’s population but face severe threats to water security, resulting in a deficit between supply and demand (Davies et al., 2016). As one of the most sensitive areas to climate change (Huang et al., 2017), challenges related to water supply are increasing (Lian et al., 2021), meaning resilient, holistic approaches to water management are crucial. One such technique, popular in certain dryland settings, is sand dams.

**Fig. 1:**
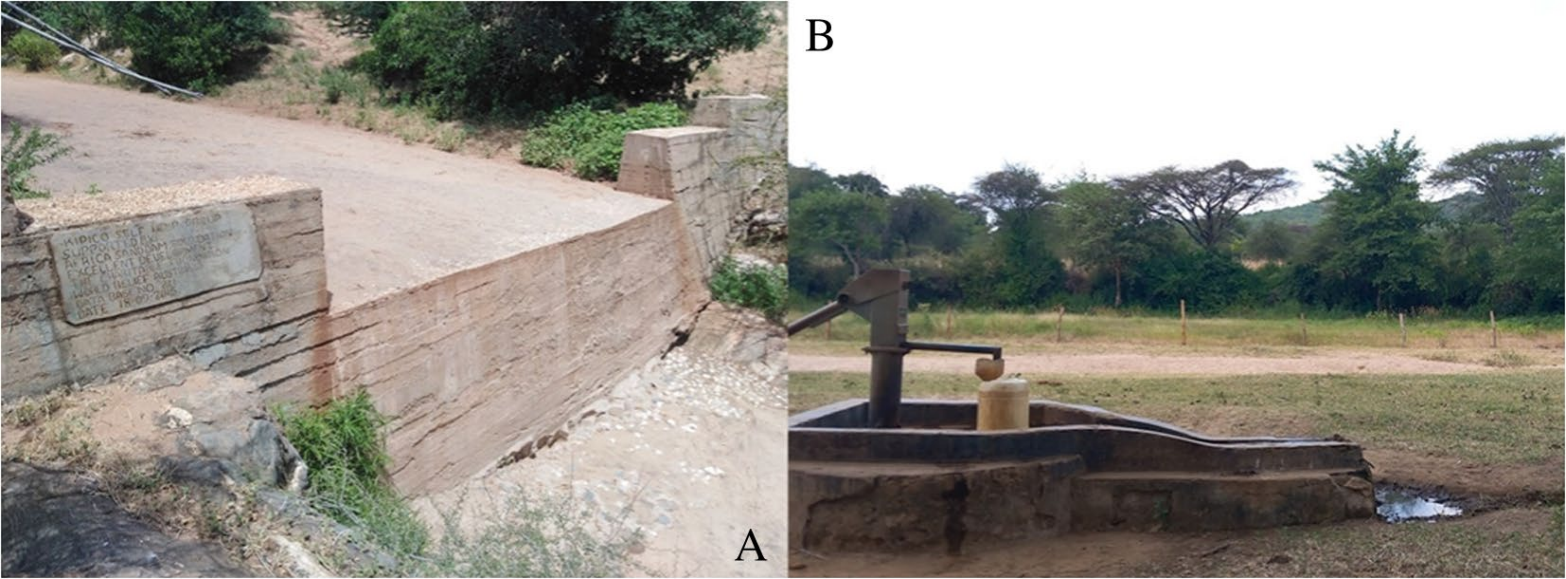
**A** Sand dam, comprising a concrete dam wall with a natural accumulation of sand behind it forming the sand aquifer; **B** handpump in the upstream bank of a sand dam

Sand dams (Fig. 1A) are small, concrete structures constructed across ephemeral streams. Alluvial sand accumulates behind the walls, in which water collects in the rainy season and is stored for use in the dry season (Neufeld et al., 2021; Pauw et al., 2008; Stern & Stern, 2011). They are indigenous structures that are found in sub-Saharan Africa, southern Africa, Asia, and North America (Ritchie et al., 2021). The water is used for domestic consumption, agriculture, livestock, and other water-consuming projects such as brickmaking (Beswetherick et al., 2018; Cruickshank & Grover, 2012; Lasage et al., 2008; Parker et al., 2022; Pauw et al., 2008; Ritchie et al., 2021; Tuinhof & Heederik, 2002). The needs of individuals using a sand dam vary, but in general the dams must rarely provide for entire year-round water needs, with other sources such as boreholes, rainwater harvesting (RWH) tanks, kiosks, surface water, springs, and piped water supply used (Lasage et al., 2013; Neufeld et al., 2020; Strohschein, 2016). Whilst people rely more on RWH tanks in the rainy season (Adekalu et al., 2002; Boelee et al., 2013; Elliott et al., 2017; Özdemir et al., 2011; Shaheed et al., 2014), sand dams can be a useful source of water in the dry season when other sources run low or are unavailable (Maddrell & Neal, 2013; Neufeld et al., 2020; Strohschein, 2016).

This region is characterised by four seasons: short dry season (January–February), long rains (March–May), long dry (June–September), and short rains (October–December) (Camberlin and Wairoto, 1997; Musyimi et al., 2023; Nganyi, 2009), with an average annual rainfall from 1981–2021 across the sites of 286 mm and 375 mm in the long and short rainy seasons respectively (derived from Climate Hazards Group InfraRed Precipitation with Station data (CHIRPS)) (CHIRPS, n.d.). Whilst the area receives reasonable rainfall, much of it is lost as runoff, leading to water deficits. According to Augustine et al. (2014), the average person in Makueni uses 19.55 +/− 0.343 L of water per day.

Sand dams are most common in Kenya, where 84% of land is arid or semi-arid and a 31% gap between available water resources and water demand is anticipated by 2030 (Davies & Gustafsson, 2015). Surface and unimproved water sources are used by 19% and 9.8% of people, respectively, endangering drinking water quality and future water security (WHO & UNICEF, 2022). However, the prevalence of streams and rivers with high coarse, suspended sediment loads offers opportunities for sand dam construction (Hut et al., 2008; Mutati et al., 2018; Viducich, 2015).

There is uncertainty around the contribution that sand dams make to year-round water security. Ngugi et al. (2020), van Loon and Droogers (2006), and de Trincheria et al. (2015) found that many dams do not have the capacity to support water needs throughout the entire long dry season in Kenya. The former two studies are located in the Tiva catchment (in the west of Kitui), whilst the latter is located in Makueni. Through surveys, Ngugi et al. (2020) found that almost 50% of 116 dams did not have water by the end of the long dry season, whilst de Trincheria et al. (2015) found that 67% of 30 dams did not supply water to even one household during the entire dry season. From modelling, van Loon and Droogers (2006) found an unmet demand from July to September. Borst and de Haas (2006), Quilis et al. (2009), and Hanson and Nilsson (1986), on the other hand, cite the opposite. The former two studies are in the Kiindu catchment (a small catchment near Kitui town (Ertsen & Ngugi, 2021)), whilst the latter is based in the Hararghe region of Ethiopia. Hanson and Nilsson (1986) and Quilis et al. (2009) cite, from measurements and modelling, respectively, that the dams are full several months after the end of the rainy season and can effectively increase water availability throughout the dry season. Borst and de Haas (2006) studied a 2-km stretch of river comprising five dams and found from a water balance that 11m^3^ is now available per day in this area from March to October, compared to 2m^3^ before dam construction, with 8m^3^ currently being used per day. Given the geographical similarity between most of the studies, location is not assumed to be the cause of differences in water availability. No studies to date have specifically quantified annual water abstraction from sand dam handpumps (Fig. 1B), creating uncertainty around the usefulness and financial viability of sand dams.

Quantifying the volume of water abstracted from sand dams via handpumps is key to understanding sand dam’s contribution to water security, and their potential resilience and sustainability against a changing climate. Whilst abstraction volumes may be linked to storage, other variables, including convenience, quality, and the use of other sources can impact abstraction. In response, this study seeks to understand the contribution that sand dams make to year-round water security by studying abstraction patterns, from sensor-derived handpump data and the characteristics of sand dams, from interviews and empirical data, which may be impacting these patterns. The study is the first of its kind at sand dam sites to use abstraction data which avoids reliance on recall and assumptions made in modelling, and which spans a continuous 31 months rather than providing a snapshot in time. It is hypothesised that sand dams improve communities’ year-round water security by sustaining water availability in the dry season. Proving or disproving this is crucial for understanding sand dams’ level of use, acceptance, and financial viability. It will help to inform future water management interventions and to ensure that users are serviced with an improved supply throughout the year.

## Materials and methods

### Study context

**Fig. 2:**
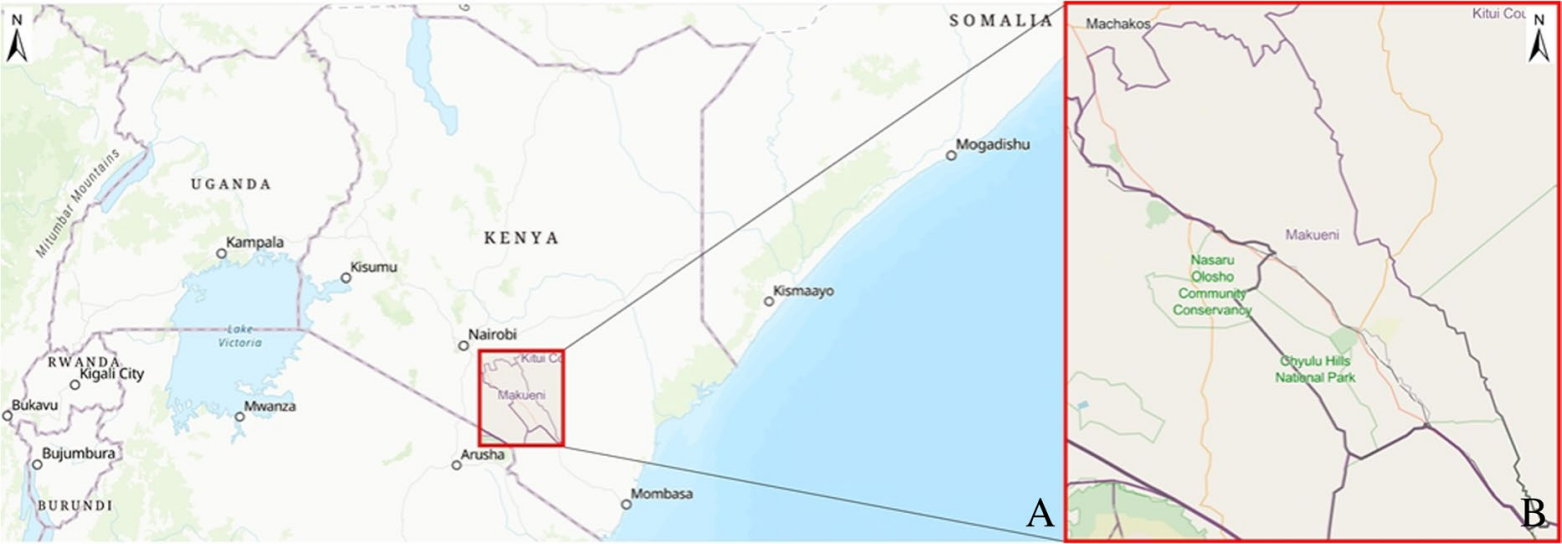
**A** Map of Kenya at a 1:9,000,000 scale, **B** map of Makueni and Machakos counties, Southeast Kenya at a 1:1,500,000 scale at A4, where the study sites are located (processed using ESRI® ArcGIS Pro^TM^)

The study location was rural areas of the Makueni and Machakos counties, in Southeast Kenya (Fig. 2), and the project was conducted in partnership with Sand Dams Worldwide (SDW) and the Africa Sand Dam Foundation (ASDF). This area was chosen due to large investment in sand dam construction.

### Data sources

To quantify the contribution that sand dam handpumps make to year-round water security, three data sets were used: sensor-derived hourly abstraction data, CHIRPS rainfall data, and interview and observational data.

The handles of thirty sand dam handpumps at thirty different sites were fitted with waterpoint data transmitters (WDT). The WDT consists of an integrated-circuit-based accelerometer, a microprocessor, and a GSM (global system for mobile communications) modem (Thomson et al., 2012). The accelerometer measures handle movement by sensing movement in X, Y, and Z planes to produce three analogue outputs proportional to the acceleration sensed along that axis. The microprocessor then takes the acceleration data in order to calculate pump handle tilt angle, which is monitored to produce a count of the number of strokes the pump handle has moved over a given time period. The relationship between stroke length and number of strokes was used to then estimate the volume of water abstracted (Thomson et al., 2012).

The data is then transmitted as a short message service (SMS) via the GSM modem (Thomson et al., 2012). Thomson et al. (2019) previously calculated the estimated volume abstracted (derived from the conversion of pump handle movements from the WTD) to give a +/− 15% accuracy against measured volume abstracted.

Each site fitted with a transmitter was selected due to (1) a strong cellular network signal so that the accelerometers can transmit data (which are converted to abstraction volume) through the mobile network and (2) the handpumps being used daily in order to provide continuous data (Chan, 2019). ASDF visited each site to verify daily usage; however, many areas with high usage had no network coverage, meaning that some sites with lower daily usage with network coverage were selected. Twenty-six of these handpumps were selected for this study based on data availability and continuity. The data spans from April 2019 to October 2021.

CHIRPS data, a 35+-year quasi global rainfall data set, was used to assess rainfall levels at the 26 dams. CHIRPS combines data from models, real-time observing meteorological stations, and 0.05° (5km) resolution infra-red satellite data to estimate daily precipitation (Funk et al., 2015). Mulungu and Mukama (2023) evaluated the accuracy of CHIRPS, finding it to correlate well with ground rainfall observations on a monthly timescale, but less well on a daily scale with Pearson’s correlation coefficients of *r*>0.7 and *r*>0.5 respectively. Although CHIRPS data is less accurate in dry than moist conditions, with light precipitation events found to be underestimated and heavy precipitation events overestimated, it has been found to be more suitable than other products for small-scale studies (Bai et al., 2018; Liu et al., 2019).

Characteristics of each dam were collected during fieldwork by Chan (2019) and provided by ASDF and SDW. Interviews and measurements were conducted at 30 sand dam communities, chosen due to the presence of a WDT (26 of which abstraction data are available for), from June 20 to July 12, 2019 (Appendix A). This part of the study gained ethical approval through the Cranfield University Research Ethics System (CURES) CURES/8640/2019. Subsequently, 203 semi-structured interviews were conducted with the assistance of a translator, in the homestead or at the handpump. Participants were selected for interview using a snowball sampling method, on the basis that they used the handpump in question. Questions were centred around volume usage and ease of access to handpumps. The interviews were intended to identify the frequency of handpump usage and to determine which factors may cause people to use other water sources over handpumps. Water samples were taken at each handpump using a plastic cup, in which electrical conductivity was tested using a hand-held probe.

Variables collected in fieldwork included perceived salinity (% of interviewees perceiving water as saline), abstraction limits (inc. only selling to members, limited number of jerry cans/p/day), livestock use (% of interviewees using the handpumps for livestock), whether the dam is said to have ever run dry, presence of rainwater harvesting tanks (% of interviewees with tanks), and actual salinity (μs/cm). These variables were chosen based on knowledge regarding possible reasons for the use of such wells (Chan, 2019). Variables provided by ASDF and SDW included area of vertical face of dam wall (area: height × length of wall (m^2^)), distance (average distance of round trip to handpump (km)), and user numbers. These were analysed using two models to provide insight into differences in abstraction between sites.

### Data analysis

The rainfall data was analysed in Google Earth Engine (Gorelick et al., 2017). A single CHIRPS cell was selected for the coordinates of each dam to give a time series of daily rainfall data across the study period. The data from each site was averaged across the study period and from 1981 to 2021 to also give long-term average rainfall.

Some abstraction data was missing due to signal and battery failures. Hourly abstraction of 0L (handpumps not used) was differentiated from missing data, which was represented by NA or an excluded data point. The raw hourly data was collated into daily, weekly, monthly, and seasonal data for the analysis.

To account for the missing data, all days with any missing hourly data were excluded so that total daily abstraction was based on a complete day. Following this, specific inclusion criteria were developed for different abstraction data sets depending on the analysis:

- To analyse patterns of abstraction, daily, monthly, and seasonal (L/day) data sets were used. All data were used, apart from four outliers at Wikwatyo of >14,000L/day which were removed where necessary for clarity in figures.
- To analyse the relationship between sand dam characteristics and abstraction volume, median monthly abstraction per person (L/person/day) was used. Only handpumps with data for all variables in the models were included.
- To analyse abstraction patterns in the long dry season, median weekly abstraction per person (L/person/day) was used. Only handpumps with >4 days’ worth of data available for the last week of September in at least 1 year were used.

These data sets were used to assess the contribution that each handpump was making to year-round water security. The data were coded and analysis performed in R Studio (RStudio Team, 2020) and Stata (StataCorp, 2021).

Descriptive and inferential statistics were used to explore general patterns of abstraction, both spatially and temporally. A Friedman test, a non-parametric alternative to a one-way ANOVA test, used when measurements of a group of subjects are taken over time, was used to assess statistical difference in the median daily abstraction between seasons (L/day). Following a detrending of the data, total monthly abstraction was compared to total monthly rainfall at each site, using Spearman’s rank correlations (non-parametric version of a Pearson test), to assess whether monthly abstraction varies significantly with rainfall.

To explore which characteristics may impact abstraction, two random effects regression models were used, as such models can handle panel data and are used to fit a relationship between two or more variables (Park, 2011). A Hausman test indicated the merits of a random over a fixed effect model, and the dependent variable (median monthly abstraction (L/day)) was log transformed, enabling assumptions of a linear model to be met. Median rather than mean abstraction was used due to the skewness of data. A monthly timescale was used to assess seasonal variation over the year. Significant (*p*) independent predictors of outcomes were assessed at *p* < 0.05, <0.01, and <0.001. The first model included the independent variables: month, site, actual salinity, area of dam wall, livestock usage, abstraction limits, and the proportion of interviewees with a RWH tank. The second model additionally included distance. These variables were chosen for exploration purposes and due to previous research findings (actual salinity, livestock use, RWH tank, and distance (Chan, 2019; Kelly et al., 2018; Rahman et al., 2017; van Schalkwyk, 1996)). Variables were dummy coded as categorical variables into two groups of roughly even sizes centred around the average.

Finally, analysis in the last full week of September (end of the long dry season) assessed whether handpumps were providing at least sufficient drinking water by this point. Due to the necessity of an improved source of water for drinking, the handpumps were classified based on a median weekly abstraction of 2L/p/day and 4L/p/day in the final full week of September, based on the minimum drinking water requirement cited by the World Health Organisation (2016) in regular and hot climates respectively. This was done to assess whether the handpumps could independently meet drinking water needs, in case no other water source was available. The handpumps were sorted into three groups based on a median weekly abstraction of 2L/p/day (the lower cited minimum drinking water requirement (World Health Organisation, 2016)). Due to the 15% uncertainty in the data, the groups were as follows: <1.7L/p/day not providing sufficient water, 1.7–2.3L/p/day potentially providing sufficient water, >2.3L/p/day providing sufficient water. Any handpumps providing >4.6L/p/day, derived from the minimum drinking requirement in hot climates (World Health Organisation, 2016) with a 15% uncertainty, are marked with an X in Table 3. Values were based on per person abstraction using an average of 3.7 residents per household (Knoema, 2019). However, due to differences in cited household size, with Augustine et al. (2014) citing an average of 6.15 residents per household in Makueni, any handpumps with >2.3L/p/day still being abstracted when using a household size of 6.15 are marked with a 0 in Table 3.

## Results

### Rainfall

Average monthly rainfall across the 26 study sites for the duration of the study period (April 2019–October 2021) displayed the recognised Kenyan trend of two rainy and two dry seasons. Long-term average yearly rainfall across the 26 study sites from 1981 to 2021 was 760mm. Yearly rainfall for 2019, 2020, and 2021 was 1035mm, 996mm, and 726mm, respectively, highlighting 2019 and 2020 as wetter than average (CHIRPS, n.d.).

However, on a seasonal level (based on literature derived seasons), the long rainy seasons in 2019 and 2021 were drier (160 mm and 264 mm respectively) than the long term average (286 mm), whilst 2020 was wetter (438 mm). This is in comparison to the short rainy season, which in 2019 was over two times greater (797 mm) than the average (375 mm), whilst 2020 and 2021 were slighlty drier (332 mm and 369 mm respectively) (CHIRPS, n.d.).

### Patterns of daily abstraction

#### Spread of daily data

Across the months in which each handpump transmitter was live, the amount of missing daily data ranged from 5.8% at Woni 15 to 75.4% at Woni 16. Seven handpumps had >50% of data missing, whilst twelve had <25% of data missing. Not all transmitters were operational from April 2019 to October 2021, making it an unbalanced data set. The data set of daily abstraction showed a non-normal distribution, with a high positive skew to the right (skewness = 3.58, indicating a high proportion of small values). Of 11,610 data points, there were *n*=840 counts of 0L abstraction, whilst most values (1,307) lay between >0 and 250L/day.

#### Heterogeneity between sites

**Fig. 3:**
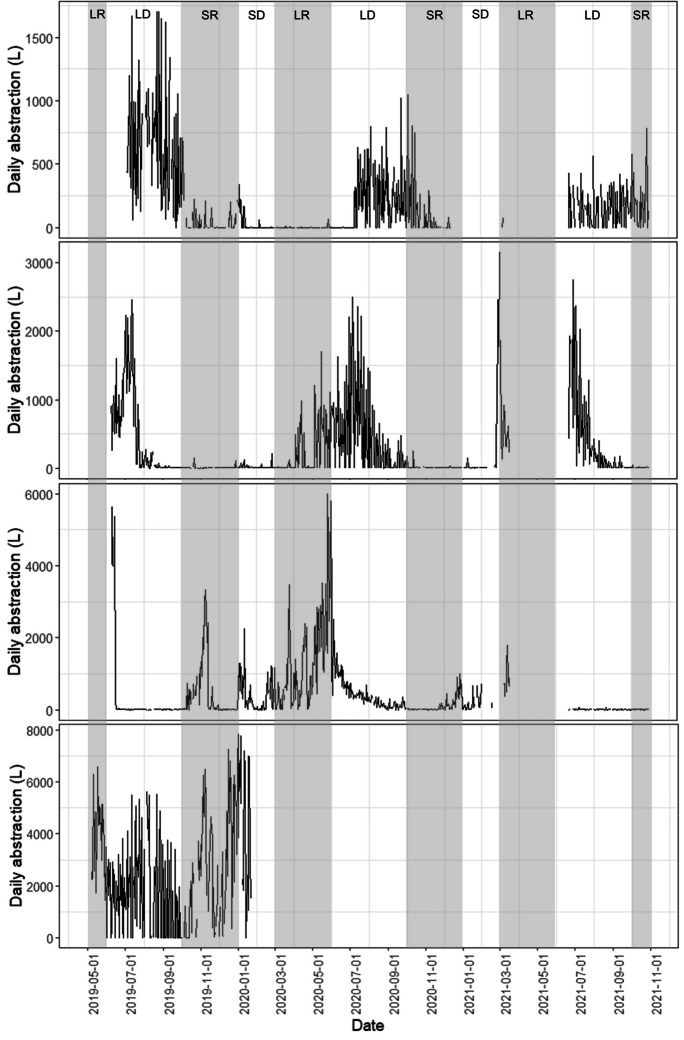
Daily time series of abstraction at sites **A **Ikanga, **B** Mapatano 18, **C** Makutano, and **D** Woni 15. Rainy season is shaded. LR, long rainy; LD, long dry; SR, short rainy; SD, short dry season

**Fig. 4:**
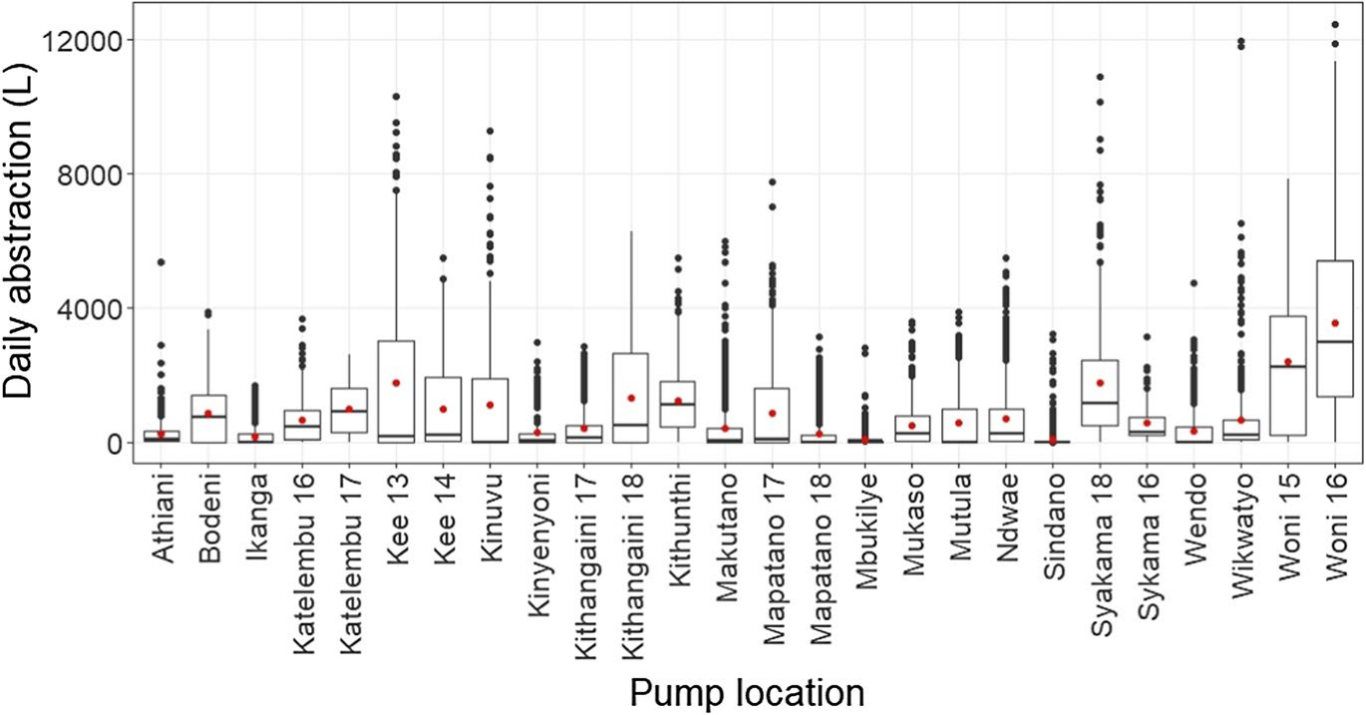
Daily abstraction from each site across the study period (red dot shows the mean value compared to the median line). Four values at Wikwatyo of >14,000 L have been removed

There was heterogeneity in daily abstraction patterns and volumes between handpumps. Four sites (Fig. 3) have been used to represent the main patterns observed. Most handpumps saw peaks of abstraction in the long dry season (*n*=17) (Fig. 3A), whilst some also saw peaks in the short dry season (*n*=5) (Fig. 3B). Others saw additional peaks in the rainy season (*n*=11) (Fig. 3C), whilst some showed similarly high levels of abstraction across the year with no distinct peaks (*n*=3) (Fig. 3D). Due to this heterogeneity between handpumps, median daily abstraction, although 95L overall, varied greatly between individual sites from 2L at Ikanga to 2962L at Woni 16 (Fig. 4).

#### Heterogeneity between seasons

**Fig. 5:**
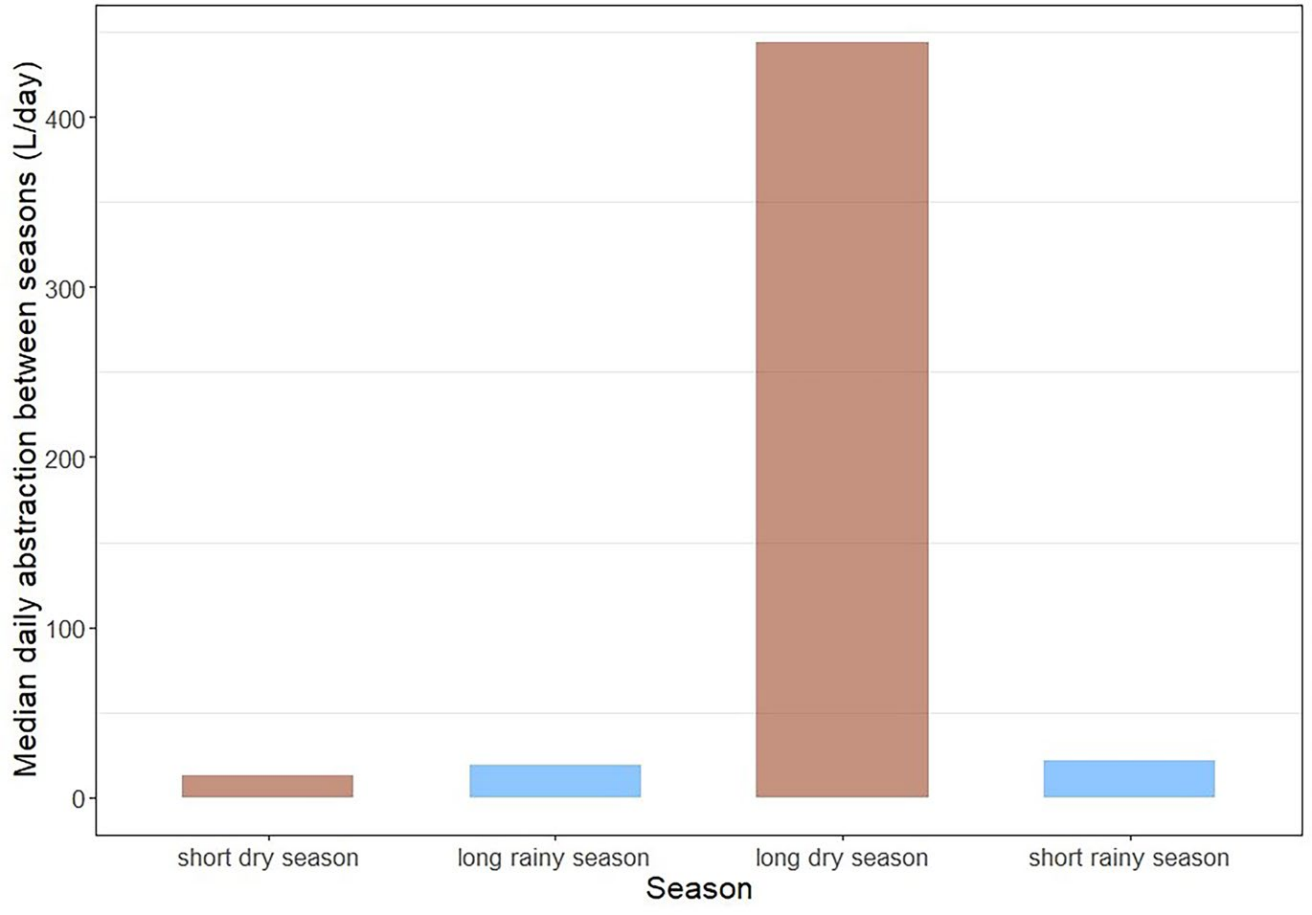
Median daily abstraction between seasons (L/day) across all sites

**Fig. 6:**
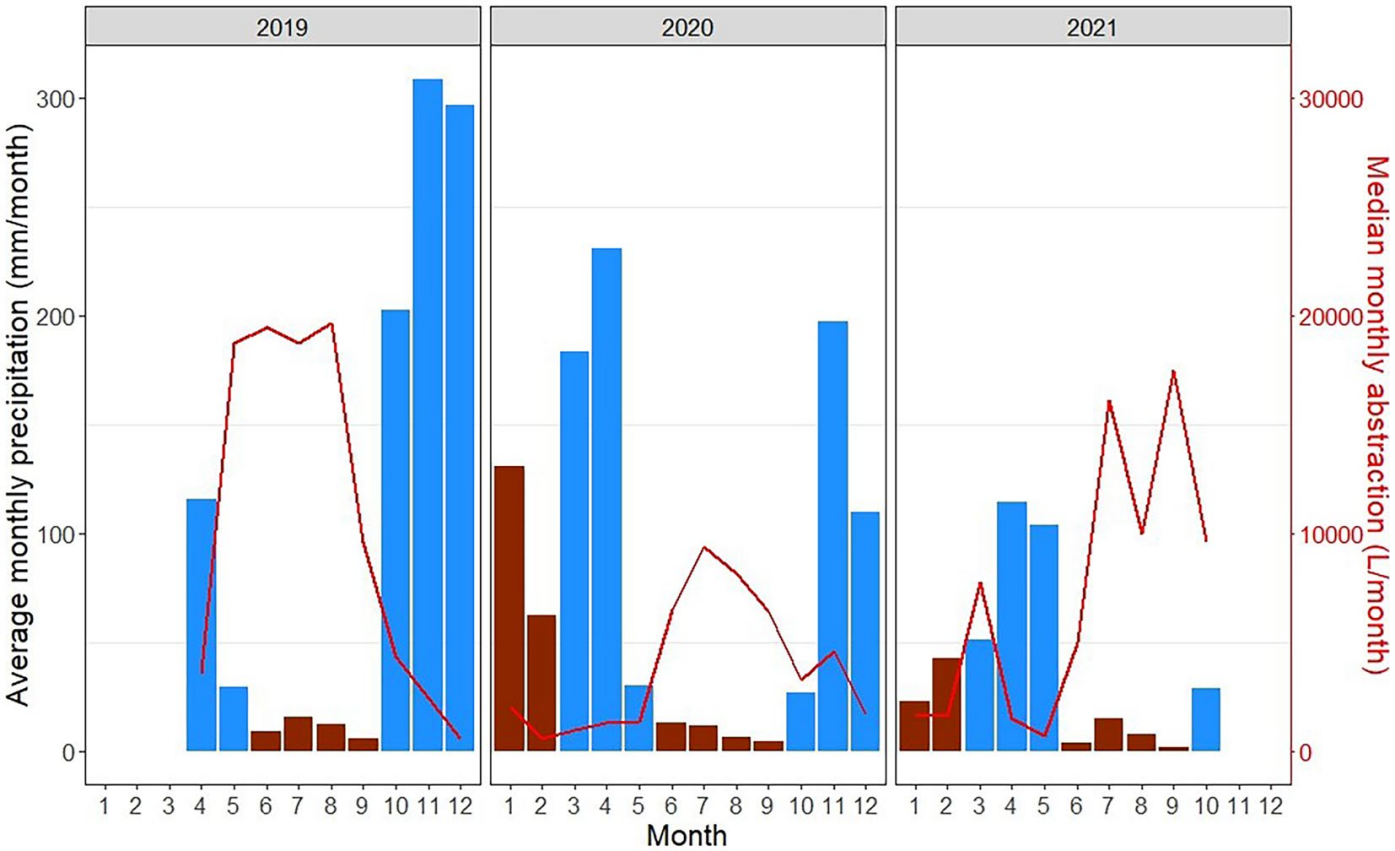
Median monthly abstraction (L/month) (red line) and average monthly precipitation (mm/month) (brown bars — dry season and blue bars — rainy season) across all sites

Handpump use was heterogenous between seasons (Figs. 5 and 6). Whilst the range of abstraction data across the handpumps was not dissimilar in each season, the median abstraction was highest in the long dry season (444 L/day) compared to 13 L/day in the short dry season (Fig. 5). A Friedman Test indicated a significant (*p* = 0.0145) difference in median daily abstraction between seasons (L/day) amongst individual sites.

Median monthly abstraction averaged across all sites showed a peak in the long dry season (August in 2019, July in 2020, and September in 2021). There is generally a decreasing trend in abstraction towards the end of the long dry season in 2019 and 2020. Lower levels of abstraction were observed throughout the rest of the year, with the lowest occurring at the end of the short rainy season in 2019, in the short dry season in 2020, and at the end of long rainy season in 2021 (Fig. 6). Spearman’s rank correlations of total monthly abstraction compared to total monthly rainfall at each individual site indicated a significant negative correlation at four sites and a positive correlation at one: Athiani (*r*=.45, *p*=0.038), Kithunthi (*r*=−.52, *p*=0.007), Mapatano 17 (*r*=−.48, *p*=0.010) Mbukilye (*r*=−.64, *p*=0.0003), and Wendo (*r*=−.74, *p*=2.15e-05), with the four latter sites indicating significantly higher monthly abstraction when rainfall is lower. Most of the other sites also show negative correlations, as shown in Fig. 6, displaying median monthly abstraction and precipitation across each site.

### Relationship between sand dam characteristics and abstraction volume

In order to assess whether any sand dam characteristics or individual behaviours were influencing abstraction, two random effects regression models were used. Table 1 highlights the descriptive statistics of the independent variables of sand dam characteristics or individual behaviour: actual salinity, area of vertical face of dam wall, livestock use, abstraction limits, presence of rainwater harvesting tanks, distance, whether the dam is said to have ever run dry, and perceived salinity, alongside the dependent variable (median monthly abstraction per person), and the control variables month and handpump site.

**Table 1:** Selected dependent variable characteristics used in the regression models

Description	Value	Number of data points	Mean	Median	Min	Max
Dependant variable						
Median monthly abstraction per person (L/day)	0–67.5	496	5.2	1.1	0	67.5
Categorical variables						
Actual salinity (μs/cm)	0 (<1000) 1 (1000+)	254 215	1218	938	208	3150
Area of dam wall (m^2^) — height × length of wall	0 (<90) 1 (90+)	356 117	105.9	72	36.6	420
Livestock usage (%) — % of interviewees using the handpumps for livestock	0 (<40) 1 (40+)	228 212	40.7	33	0	100
Abstraction limits (inc. only selling to members, limited number of jerry cans/p/day)	0 (no) 1 (yes)	151 289	0.7	1	0	1
Rainwater harvesting tank (%) — of interviewees with tanks	0 (<42) 1 (42+)	245 172	43.6	40	0	83
Distance (km) — average distance of round trip to handpump	0 (0.5) 1 (>0.5)	185 166	0.7	0.5	0.5	2.1
Ever run dry	0 (no) 1 (yes)	275 44	0.1	0	0	1
Perceived salinity (%) — % of interviewees perceiving water as saline	0 (<60) 1 (60+)	161 253	50	50	0	100
Control variables						
Month		31			04. 2019	10. 2021
Pump		26				

Data on five of the independent variables of sand dam characteristics or individual behaviours (Table 1): actual salinity, area of wall, livestock, abstraction limits, and RWH tank, whilst controlling for month and pump, were compared to log median monthly abstraction per person (L/day) in random effects regression model 1 (Table 2). Perceived salinity was excluded due to high collinearity with actual salinity. Ever run dry and distance were removed due to the loss of data due to 8 and 9 handpumps respectively having no values for these variables.

**Table 2:** Results of the regression analysis

	Model 1	Model 2
Control variables		
Actual salinity	2.489***	1.864**
Area of wall	4.132***	3.166***
Livestock use	1.252**	0.800
Abstraction limits	0.713	1.748*
RWH tank	0.788	−0.368
Distance	-	−0.955
Observations	390	284
Groups	22	16
*R*^2^	0.471	0.500
Wald chi^2^	301.44	237.88
Max VIF	1.88	2.76

*p < 0.05; **p < 0.01; ***p < 0.001

This first model explained 47.1% of the variation in abstraction (28.1% within and 100% between). Within variation represents the total variation in the individual values in each group and their group mean whilst between variation represents the total variation between each group mean and the overall mean.

Except for September 2019, June 2020, and July 2020, 75% of the long dry months during the 3 years were found to have significantly higher abstraction than the reference month of January 2020.

Abstraction was also significantly higher in the rainy seasons of May 2019, Oct 2020, and October 2021. Sites where actual salinity was higher, dam wall area was larger, and more people were using the water for livestock had significantly higher levels of abstraction. Abstraction limits and the proportion of people with RWH tanks were not significant.

Distance was included in a second model, using the reduced number of handpumps (Table 2). Ever run dry was still excluded as its inclusion meant that the requirements for a random effects regression model were not met. The second model explained 50.0% of the variation in abstraction (27.0% within and 100% between sites). Of the long dry months across the 3 years, 41.7% significantly determined abstraction, with abstraction being significantly higher from the reference month of January 2020. Abstraction was also significantly higher in March 2021 and Oct 2021. Sites where actual salinity was higher, dam wall area was larger, and abstraction limits were in place had significantly higher levels of abstraction. Livestock use, the proportion of people with RWH tanks, and distance were not significant. As distance was not significant in model 2 and given the reduced number of sites used, the first model is the focus of the discussion in the “Temporal abstraction trends” section.

### Long dry season analysis

Of the 26 handpumps, 22 had abstraction data available for the last full week of at least one long dry season (September), with 21 having some water abstracted, indicating that they were providing water when access to other sources may have been compromised. Median daily abstraction in this week averaged across the 3 years across the 22 sites was 242L. This varied, however, between 0L at Bodeni and 5920L at Kee 13.

From the 22 handpumps for which data was available, using a household size of 3.7, 31.8% (*N*=7) of sites were classed as providing sufficient water (>2.3L/p/day) by the end of each long dry season (2019–2022) for which they have data available: able to meet users’ minimum drinking needs. A further 27.3% (*N*=6) met this need by the end of at least one long dry season for which they have data available. Of these 13 handpumps, 84.6% (*N*=11) were providing >4.6L per person: the drinking water needs in hot climates (World Health Organisation., 2016) in at least one long dry season (indicated by an X in Table 3). These handpumps were able meet user’s drinking water needs independently of other sources (blue in Table 3).

**Table 3:**
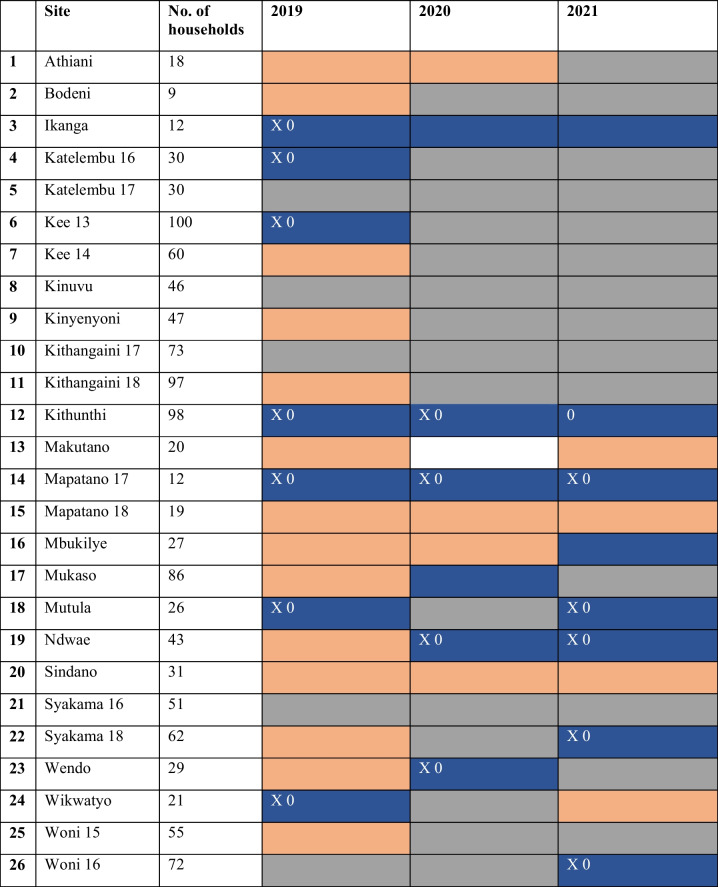
The handpumps and years where minimum user drinking needs are and are not being met by the last full week of the long dry season (September) based on median weekly abstraction (L/p/day) using an average household size of 3.7 (dark blue = >2.3L/p = providing sufficient water, brown = <1.7L/p = not providing sufficient water, white = potentially providing sufficient water (1.7–2.3L/p), grey = missing data, X = providing >4.6L/p, 0 = providing >2.3L/p when using a household size of 6.15)

Of the remaining handpumps, 36.4% (*N*=8) were not providing sufficient water (<1.7L), failing to meet the drinking water needs of their users in any year (brown in Table 3). One handpump (Makutano) was classed as “potentially providing sufficient water” in 2020 (between 1.7 and 2.3L) (white in Table 3).

Finally, of the 21 seasons in which >2.3L was being abstracted using a household size of 3.7, 81% (*N*=17) still had >2.3L being abstracted when using a household size of 6.15 (indicated by a 0 in Table 3), indicating that the majority would still have been providing sufficient water to meet drinking water needs even with a larger user population.

## Discussion

The contribution that sand dam handpumps make to water security is fundamental to their practicality and usefulness as a water resource in drylands. It was hypothesised that sand dams improve communities’ year-round water security by providing water from handpumps in the dry season. As the first to analyse a long-term timeseries of abstraction data from sand dams, this study supports this hypothesis in general; however, heterogeneity between sites and missing data do not make general conclusions possible.

### Temporal abstraction trends

Abstraction from the handpumps is highly heterogenous between seasons. When rainfall is higher, utilisation of handpumps is generally lower. This is consistent with the findings of Thomson et al. (2019) who studied the relationship between rainfall and groundwater use in Kwale County on Kenya’s southern coast. They found highest weekly abstraction (average 2335L/pump/day) to be the first week of March, at the end of the short dry season. This was three times greater than that of the second week of May (average of 789L/pump/day), around the peak of the rains. This is consistent with the regression model in this study which found significantly higher abstraction in 75% of the long dry season months. It is possible that the lack of significance in the two months in 2020 was because of a wetter than average long rainy season, meaning that other surface water sources were more readily available.

Lower levels of abstraction observed in months outside of the dry season are hypothesised to be linked to the recorded increase in use of multiple sources in the rainy season (Arouna & Dabbert, 2010; Elliott et al., 2017; Kelly et al., 2018). Despite the assumed availability of water in the sand dams in the rainy season, people may choose to use other sources for numerous reasons such as cost, labour, and convenience, the latter of which is often cited as the most influential (Dessalegn et al., 2013). Many sources, however, are compromised in the dry season, forcing use of alternative sources (Chan, 2019; Kaptué et al., 2013; Kelly et al., 2018; van Houweling, 2016), likely explaining why sand dam handpump abstraction increases.

This study found a 34-times increase in median daily abstraction between seasons (L/day) from the sand dam handpumps from the short dry to the long dry season. Neufeld et al. (2020) found abstraction from sand dams in the Ukambani region of Kenya to increase from around 50 to 70% of households from the rainy to dry season, whilst RWH tank usage fell from >80 to just 10%. Thomson et al. (2019) similarly found that 86% of households used handpumps as their main drinking water source in the dry season compared to 6% in the rainy season, whilst participants interviewed for this study reported to fetching less or no water from handpumps when their rainwater harvesting tanks are full. Understanding the response of sand dams to rainfall highlights their resilience to future changes in climate and their applicability in drought-prone areas (Macdonald et al., 2019). By providing insight into when abstraction is lower, this study can help to inform water providers on times of the year when water supply from other sources such as vendors may need expanding.

### Heterogeneity in abstraction

The results show high heterogeneity in abstraction between handpumps, with median daily abstraction ranging from 2 to 2962L. Possible reasons for this heterogeneity include water availability due to sand dam characteristics, including area of wall, grain sizes, depth of sediment, width of riverbed, and slope of banks (Aerts et al., 2007; Borst & de Haas, 2006; de Trincheria et al., 2015; Jansen, 2007; Nissen-Petersen, 2006; Quinn et al., 2019; Strohschein, 2016) and environmental characteristics, including rainfall, soil, geology, accessibility, topography, and stream order (Jeil et al., 2020; Ngugi et al., 2020; Thwala, 2010; Wrisdale et al., 2017; van Houweling, 2016). Additionally, social factors including number of users, average distance to each dam, quality, safety, availability and use of other sources, perceptions, volume abstraction limits, tradition, price, and water use practices (particularly whether livestock drink directly from pooled water or if water is fetched for them from the handpump) may impact use (Chan, 2019; Devoto et al., 2012; Elliott et al., 2017; Howard, 2002; Jiang & Rohendi, 2018; Parameswari et al., 2016; WHO & UNICEF, 2017).

Modelling of sand dam characteristics against abstraction volume goes some way to explaining the variation seen across sites. In the regression model, abstraction was significantly higher at sites with higher actual salinity, larger dam walls, and where a greater proportion of interviewees use the water for livestock.

#### Actual salinity

Abstraction was significantly higher at sites with higher salinity (>1000 μs/cm). The WHO (2017) permissible level of electrical conductivity for drinking water is 2500 μs/cm, of which 12% (*N*=3) of samples exceeded. People often seek alternative sources to using saline water for drinking (Kinniburgh & Smedley, 2001) due to perceived health implications, such as hypertension, (pre)eclampsia and infant mortality (in pregnant women), respiratory infection, and kidney disease (al Nahian et al., 2018; Rutherford et al., 2015; Shammi et al., 2019). As only a small part of daily water use is used for drinking, it was discovered that abstraction was higher at locations used to meet other needs, with individuals continuing to use saline water for purposes other than drinking. Despite measured salinity being higher at Woni 16, this handpump was used more than Woni 15, with people continuing to use Woni 16 for livestock and domestic use, compared to Woni 15, which was used for cooking and drinking.

#### Dam wall area

At 52% of sites, the dam wall has an area of 90m^2^ and above (Table 1), at which abstraction was significantly higher. This is likely due to a greater volume of sand being able to build up behind a larger dam wall, and therefore a greater volume of water being stored (Borst & de Haas, 2006; Jansen, 2007; Maddrell & Neal, 2012; van Loon & Droogers, 2006).

#### Livestock

At 45.8% of sites, >40% of interviewees were using the handpump water for livestock (Table 1). These sites experienced significantly higher abstraction. When comparing interviewees’ self-reported abstraction volumes to livestock usage, it was found that those using the pumps for livestock were abstracting 30.6% more water. It is estimated that a minimum of 7.5L/p/day is required to meet basic domestic water needs (Howard & Bartram, 2003), compared to higher requirements for livestock, with growing heifers of 182 kg in 32.2 °C having an approximate daily water intake of 36 L (Subcommittee on Beef Cattle Nutrition et al., 2000).

#### Variables that do not predict abstraction volume

Abstraction limits did not significantly predict abstraction in the model, suggesting their lack of impact on water use. Sand dam users on the ground can assess the availability of water from the dams to a much better extent than a model can do and should and will continue to do what they consider necessary to conserve and sustain water resources.

Finally, the lack of significant impact of RWH tanks on abstraction suggests people’s reliance on the handpumps independently of RWH tanks. This is likely because RWH tanks are used to a greater extent in the rainy season (Adekalu et al., 2002; Boelee et al., 2013; Elliott et al., 2017; Özdemir et al., 2011; Shaheed et al., 2014), compared to sand dam handpumps which are used more in the dry season, indicating that a holistic view of rainwater harvesting needs to be taken, considering all available options as one managed whole.

### Drinking water needs

Of the 22 handpumps with available data, 21 had some water abstracted by the last week of the long dry season (median daily abstraction of 242L), indicating a contribution to year-round water security. This is consistent with Hanson and Nilsson (1986) who cite that in Bombas, Ethiopia, “the dam is full several months after the end of the rainy season” and Quilis et al. (2009) who claim that “sand-storage dams can effectively increase water availability throughout the dry season”. Whilst this is true, the level to which they can meet water needs independently of other sources is questionable at some sites due to high heterogeneity in handpump abstraction.

The results from Table 3 clearly demonstrate that the level of abstraction is not sufficient to support the drinking needs of all users of all handpumps in all years. This heterogeneity is consistent with the findings of Ngugi et al. (2020), who used a water requirement calculation using number of households, persons per household, daily per capita water requirements, and number of days in the long dry season, followed by a survey to assess whether dams were meeting these needs. They found that only 42% of dams could meet household water demand throughout the whole long dry season, whilst our study found that 59.1% were meeting drinking water needs by the end of at least one long dry season.

The variation in abstraction between years shows that whilst some handpumps can provide 2L/person/day, other factors may be impacting abstraction such as the availability of other sources, population numbers which may not remain stable, variable rainfall rates, and the maturity of the dam. The lack of data for some sites also means that further conclusions cannot be drawn.

Whilst sites were selected due to a known reasonable signal, across the period that each transmitter was live there is some missing data. Uneven proportions of missing data in different seasons and sites place limitations on the comparisons that can be made between sites and time periods. This is an inherent challenge in certain, especially rural settings across the world. This study in part overcame this uncertainty by using average values and developing exclusion criteria. It can also be argued that the benefits of using WDT over or in coordination with traditional methods of data collection outweigh the uncertainties. Using this data, the study has positively contributed to the literature on sand dams. Whilst there is heterogeneity in abstraction between seasons and sites, many handpumps are still being used by the end of the long dry season, with significantly more abstraction seen at sites with higher salinity, larger dam walls, and higher livestock usage. Sand dams can therefore be seen as an effective dryland water source, especially useful in the dry season.

## Conclusion

As the first study to analyse multi-year abstraction trends from sand dam handpumps, it reveals, quantitatively, the positive contribution that sand dams can make to year-round water security, by providing a water source by the end of the long dry season when other water sources are compromised. Of 22 handpumps with abstracted data for the end of at least one long dry season, 59.1% suggest they can meet drinking water needs by the end of the long dry season independently of other sources.

Having said this, heterogeneity in abstraction is a reminder that not all sand dams behave the same, with certain sand dams always likely to have higher levels of abstraction than others, such as those with higher user numbers, levels of salinity, livestock use, and larger dam walls. The findings of low abstraction from certain handpumps and in certain months challenge sweeping statements in literature professing the success and perfection of all sand dams, which are neither accurate nor helpful. Yet, as abstraction levels are not synonymous with water storage in the dam, with many confounding factors impacting level of abstraction, without further research, their true capabilities are unknown. High abstraction and sustained water availability by the end of the long dry season at many sites profess the positive contribution that sand dams can make to a community’s water supply, offering opportunities for further success in the future.

### Supplementary information

ESM 1 (DOCX 180 kb)

## Data Availability

The data sets analysed during the current study are available in the Cranfield University repository (CORD) at 10.17862/cranfield.rd.21435690.

## References

[CR1] Adekalu KO, Osunbitan JA, Ojo OE (2002). Water sources and demand in South Western Nigeria: Implications for water development planners and scientists. Technovation.

[CR2] Aerts J, Lasage R, Beets W, de Moel H, Mutiso G, Mutiso S, de Vries A (2007). Robustness of sand storage dams under climate change. Vadose Zone Journal.

[CR3] al Nahian, M., Ahmed, A., Lázár, A. N., Hutton, C. W., Salehin, M., & Streatfield, P. K. (2018). Drinking water salinity associated health crisis in coastal Bangladesh. *Elementa, 6*. 10.1525/elementa.143

[CR4] Arouna A, Dabbert S (2010). Determinants of domestic water use by rural households without access to private improved water sources in Benin: A seemingly unrelated Tobit approach. Water Resources Management.

[CR5] Augustine AO, Afullo AO, Danga BO, Odhiambo F (2014). Implications of time to water source on water use in the arid and semi-arid land counties of Kenya. International Journal of Water.

[CR6] Bai, L., Shi, C., Li, L., Yang, Y., & Wu, J. (2018). Accuracy of CHIRPS satellite-rainfall products over mainland China. *Remote Sensing, 10*(3). 10.3390/rs10030362

[CR7] Beswetherick, S., Carrière, M., Legendre, V., Mather, W., Perpes, T., Saunier, B., Pablo, S., Moreno, C., Pidou, K. L. C., & Parker, A. (2018). Guidelines for the siting of sand dams [Unpublished master’s project]. Cranfield University.

[CR8] Boelee E, Yohannes M, Poda JN, McCartney M, Cecchi P, Kibret S, Hagos F, Laamrani H (2013). Options for water storage and rainwater harvesting to improve health and resilience against climate change in Africa. Regional Environmental Change.

[CR9] Borst, L., & Haas, S. A. de. (2006). Hydrology of sand storage dams a case study in the Kiindu catchment, Kitui District, Kenya. [Master’s thesis, Vrije Universiteit]. SamSam Water. Microsoft Word - L. Borst & S.A. de Haas - Hydrology of Sand Storage Dams - A case study in the Kiindu catchment, Kitui Distric (samsamwater.com)

[CR10] Camberlin P, Wairoto JG (1997). Intraseasonal wind anomalies related to wet and dry spells during the ‘long’ and ‘short’ rainy seasons in Kenya. Theoretical and Applied Climatology.

[CR11] Chan, J. (2019). Abstraction of water from sand dams in Machakos and Makueni counties (Kenya) via handpumps. [Unpublished master’s thesis]. Cranfield University.

[CR12] CHIRPS. (n.d.). CHIRPS-GEFS precipitation forecasts. 10.15780/G2PH2M

[CR13] Cruickshank A, Grover VL (2012). These are our water pipes—Sand dams, women and donkeys: Dealing with water scarcity in Kenya’s arid and semi-arid lands. Climate change and the sustainable use of water resources.

[CR14] Davies EJ, Barchiesi S, Ogali CJ, Welling R, Dalton J, Laban P (2016). Water in drylands: Adapting to scarcity through integrated management.

[CR15] Davies, W., & Gustafsson, J. (2015). Water resources in Kenya: Closing the gap. Kenya-Hydro-Economic-Briefing-Note_May2015.pdf (2030wrg.org)

[CR16] Dessalegn, M., Nigussie, L., Tucker, J., Nicol, A., & Calow, R. (2013). Voices from the source. Voices from the source: struggles with local water security in Ethiopia - - Research reports and studies (wateraid.org).

[CR17] Devoto F, Duflo E, Dupas P, Parienté W, Pons V (2012). Happiness on tap: Piped water adoption in urban Morocco. American Economic Journal: Economic Policy.

[CR18] Elliott M, MacDonald MC, Chan T, Kearton A, Shields KF, Bartram JK, Hadwen WL (2017). Multiple household water sources and their use in remote communities with evidence from Pacific island countries. Water Resources Research.

[CR19] Ertsen, M. W., & Ngugi, K. N. (2021). Ambivalent assets: The success of sand-storage dams for rainwater harvesting in Kitui County, Kenya. *Frontiers in Water, 3*). Frontiers Media S.A. 10.3389/frwa.2021.676167

[CR20] Funk C, Verdin A, Michaelsen J, Peterson P, Pedreros D, Husak G (2015). A global satellite-assisted precipitation climatology. Earth System Science Data.

[CR21] Gorelick N, Hancher M, Dixon M, Ilyushchenko S, Thau D, Moore R (2017). Google Earth Engine: Planetary-scale geospatial analysis for everyone. Remote Sensing of Environment.

[CR22] Hanson G, Nilsson A (1986). Ground-water dams for rural-water supplies in developing countries. Ground Water.

[CR23] Howard G (2002). Water supply surveillance: A reference manual.

[CR24] Howard, G., & Bartram, J. (2003). *Domestic water quantity, service level and health*. In World Health Organization. https://apps.who.int/iris/handle/10665/67884

[CR25] Huang J, Li Y, Fu C, Chen F, Fu Q, Dai A, Shinoda M, Ma Z, Guo W, Li Z, Zhang L, Liu Y, Yu H, He Y, Xie Y, Guan X, Ji M, Lin L, Wang S, Wang G (2017). Dryland climate change: Recent progress and challenges. Reviews of Geophysics.

[CR26] Hut R, Ertsen M, Joeman N, Vergeer N, Winsemius H, van de Giesen N (2008). Effects of sand storage dams on groundwater levels with examples from Kenya. Physics and Chemistry of the Earth.

[CR27] Jansen, J. (2007). The influence of sand dams on rainfall-runoff response and water availability in the semi-arid Kiindu catchment, Kitui District, Kenya. [Master’s thesis, Vrije Universiteit]. SamSam Water. https://samsamwater.com/library/Jansen_-_2007_-_The_influence_of_sand_dams_on_rainfall-runoff_response_and_water_availability_in_the_semi-arid_Kiindu_catchment_Kitui_District_Kenya.pdf. Accessed 24 Aug 2023

[CR28] Jeil EB, Abass K, Ganle JK (2020). “We are free when water is available”: Gendered livelihood implications of sporadic water supply in Northern Ghana. Local Environment.

[CR29] Jiang Y, Rohendi A (2018). Domestic water supply, residential water use behaviour, and household willingness to pay: The case of Banda Aceh, Indonesia after ten years since the 2004 Indian Ocean Tsunami. Environmental Science and Policy.

[CR30] Kaptué AT, Hanan NP, Prihodko L (2013). Characterization of the spatial and temporal variability of surface water in the Soudan-Sahel region of Africa. JGR Biogeosciences.

[CR31] Kelly E, Shields KF, Cronk R, Lee K, Behnke N, Klug T, Bartram J (2018). Seasonality, water use and community management of water systems in rural settings: Qualitative evidence from Ghana, Kenya, and Zambia. Science of the Total Environment.

[CR32] Kinniburgh D, Smedley P (2001). Arsenic contamination of groundwater in Bangladesh.

[CR33] Knoema. (2019). *Kenya Census Data, 2019. Kenya Census Data, 2019*. knoema.com

[CR34] Lasage R, Aerts J, Mutiso GCM, de Vries A (2008). Potential for community-based adaptation to droughts: Sand dams in Kitui, Kenya. Physics and Chemistry of the Earth.

[CR35] Lasage R, Aerts J, Verburg PH, Sileshi AS (2013). The role of small-scale sand dams in securing water supply under climate change in Ethiopia. Mitigation and Adaptation Strategies for Global Change.

[CR36] Lian X, Piao S, Chen A, Huntingford C, Fu B, Li LZX, Huang J, Sheffield J, Berg AM, Keenan TF, McVicar TR, Wada Y, Wang X, Wang T, Yang Y, Roderick ML (2021). Multifaceted characteristics of dryland aridity changes in a warming world. Nature Reviews Earth and Environment.

[CR37] Liu, J., Shangguan, D., Liu, S., Ding, Y., Wang, S., & Wang, X. (2019). Evaluation and comparison of CHIRPS and MSWEP daily-precipitation products in the Qinghai-Tibet Plateau during the period of 1981–2015. *Atmospheric Research, 230*. 10.1016/j.atmosres.2019.104634

[CR38] Loon, A. van, & Droogers, P. (2006). Water evaluation and planning system, Kitui – Kenya (Report No. 2). WatManSup project. Water-Bodem-Boom Relaties op Landgoed Duindigt (futurewater.nl)

[CR39] Macdonald AM, Bell RA, Kebede S, Azagegn T, Yehualaeshet T, Pichon F, Young M, Mckenzie AA, Lapworth DJ, Black E, Calow RC (2019). Groundwater and resilience to drought in the Ethiopian highlands. Environmental Research Letters.

[CR40] Maddrell, S., & Neal, I. (2012). Sand dams: A practical guide. Excellent Development. Maddrell_and_Neal_2012_Sand_Dams_a_Practical_Guide_LR.pdf (samsamwater.com)

[CR41] Maddrell, S., & Neal, I. (2013). Building sand dams: A practical guide. Excellent Development. http://www.samsamwater.com/library/Maddrell_and_Neal_2012_Sand_Dams_a_Practical_Guide_LR.pdf. Accessed 24 Aug 2023

[CR42] Mulungu DMM, Mukama E (2023). Evaluation and modelling of accuracy of satellite-based CHIRPS rainfall data in Ruvu subbasin, Tanzania. Modeling Earth Systems and Environment.

[CR43] Musyimi PK, Sahbeni G, Timár G, Weidinger T, Székely B (2023). Analysis of short-term drought episodes using Sentinel-3 SLSTR data under a semi-arid climate in lower eastern Kenya. Remote Sensing.

[CR44] Mutati K, Kitheka JU, Otieno E (2018). Sand gradation in seasonal rivers and their suitability for construction of sand dams in Kitui South, Kenya. Hydrology: Current Research.

[CR45] Neufeld DG, Muendo B, Muli J, Kanyari J (2020). Coliform bacteria and salt content as drinking water challenges at sand dams in Kenya. Journal of Water and Health.

[CR46] Neufeld, D. G., Muli, J., Muendo, B., & Kanyari, J. (2021). Assessment of water presence and use at sand dams in Kenya. *Journal of Arid Environments, 188*. 10.1016/j.jaridenv.2021.104472

[CR47] Nganyi, W. (2009). *Pigeonpea response to phosphorus fertiliser, temperature and soil moisture regimes during the growing season at Katumani and Kampi Ya Mawe in Machakos and Makueni Districts of Kenya*. University of Nairobi.

[CR48] Ngugi KNK, Gichaba CMM, Kathumo VMV, Ertsen MWM (2020). Back to the drawing board: Assessing siting guidelines for sand dams in Kenya. Sustainable Water Resources Management.

[CR49] Nissen-Petersen, E. (2006). Water from dry riverbeds. ASAL Consultants Ltd. 212.0-06WA-18985.pdf (ircwash.org)

[CR50] Özdemir S, Elliott M, Brown J, Nam PK, Hien VT, Sobsey MD (2011). Rainwater harvesting practices and attitudes in the Mekong Delta of Vietnam. Journal of Water Sanitation and Hygiene for Development.

[CR51] Parameswari K, Mudgal BV, Padmini TK (2016). Awareness on usage of contaminated groundwater around Perungudi dumpsite, Tamil Nadu, India. Environment, Development and Sustainability.

[CR52] Park, H. M. (2011). Practical guides to panel data modelling: A step by step analysis using Stata. (Tutorial Working Paper). Graduate School of International Relations, International University of Japan. https://www.iuj.ac.jp/faculty/kucc625/method/panel/panel_iuj.pdf. Accessed 24 Aug 2023

[CR53] Parker AH, Nyangoka J, Rodrigues I, Yadav B, le Corre KS, Campo P, Quinn R (2022). The multiple uses of water derived from managed aquifer recharge systems in Kenya and India. Journal of Water Sanitation and Hygiene for Development.

[CR54] Pauw, W. P., Mutiso, S., Mutiso, G., Manzi, H. K., Lasage, R., & Aerts, J. C. J. H. (2008). An assessment of the social and economic effects of the Kitui sand dams. SASOL & Institute for Environmental Studies. Microsoft Word - R-08-08.doc (adapts.nl).

[CR55] Quilis RO, Hoogmoed M, Ertsen M, Foppen JW, Hut R, de Vries A (2009). Measuring and modeling hydrological processes of sand-storage dams on different spatial scales. Physics and Chemistry of the Earth.

[CR56] Quinn, R., Rushton, K., & Parker, A. (2019). An examination of the hydrological system of a sand dam during the dry season leading to water balances. *Journal of Hydrology, 4*. 10.1016/j.hydroa.2019.100035

[CR57] Rahman MT, Rasheduzzaman M, Habib MA, Ahmed A, Tareq SM, Muniruzzaman SM (2017). Assessment of freshwater security in coastal Bangladesh: An insight from salinity, community perception and adaptation. Ocean and Coastal Management.

[CR58] Ritchie, H., Eisma, J. A., & Parker, A. (2021). Sand dams as a potential solution to rural water security in drylands: Existing research and future opportunities. *Frontiers in Water, 3*. 10.3389/frwa.2021.651954

[CR59] RStudio Team (2020). RStudio: Integrated development for R.

[CR60] Rutherford S, Chu CM, Talukder MR (2015). Salinization of Drinking Water in the Context of Climate Change and Sea Level Rise: A Public Health Priority for Coastal Bangladesh. The International Journal of Climate Change: Impacts and Responses.

[CR61] Shaheed A, Orgill J, Ratana C, Montgomery MA, Jeuland MA, Brown J (2014). Water quality risks of ‘improved’ water sources: Evidence from Cambodia. Tropical Medicine and International Health.

[CR62] Shammi, M., Rahman, M. M., Bondad, S. E., & Bodrud-Doza, M. (2019). Impacts of salinity intrusion in community health: A review of experiences on drinking water sodium from coastal areas of Bangladesh. *Healthcare (Switzerland), 7*(1). 10.3390/healthcare7010050 10.3390/healthcare7010050 PMC6473225 30909429

[CR63] StataCorp (2021). Stata statistical software: Release 17 (no. 17).

[CR64] Stern, J. H., & Stern, A. (2011). Water harvesting through sand dams. Echo Development Notes. Stern-2011-Water-Techn.note.pdf (ircwash.org)

[CR65] Strohschein, P. M. (2016). Exploring the influence of sand storage dams on hydrology and water use [Master’s thesis, TU Delft]. TU Delft Repository. http://resolver.tudelft.nl/uuid:ae7390ad-5884-4c4f-88de-1c6be6fc6be0

[CR66] Subcommittee on Beef Cattle Nutrition, Committee on Animal Nutrition, Board on Agriculture, National Research Council (2000). Nutrient requirements of beef cattle.

[CR67] Thwala WD (2010). Community participation is a necessity for project success: A case study of rural water supply project in Jeppes Reefs, South Africa. African Journal of Agricultural Research.

[CR68] Thomson P, Bradley D, Katilu A, Katuva J, Lanzoni M, Koehler J, Hope R (2019). Rainfall and groundwater use in rural Kenya. Science of the Total Environment.

[CR69] Thomson P, Hope R, Foster T (2012). GSM-enabled remote monitoring of rural handpumps: A proof-of-concept study. Journal of Hydroinformatics.

[CR70] Trincheria, J. de, Petersen, E., Filho, W., & Ottorphol, R. (2015). *Factors affecting the performance and cost efficiency of sand storage dams in South Eastern Kenya*. E-Proceedings of the 36th IAHR World Congress, Hague, Netherlands. https://www.iahr.org/library/infor?pid=7777

[CR71] Tuinhof A, Heederik JP (2002). Management of aquifer recharge and subsurface storage: Making better use of our largest reservoir. Water Partnership Program (WPP).

[CR72] van Houweling E (2016). “A good wife brings her husband bath water”: Gender roles and water practices in Nampula, Mozambique. Society and Natural Resources.

[CR73] van Schalkwyk, A. (1996). Report to the Water Research Commission: Guidelines for the estimation of domestic water demand of developing communities in the Northern Transvaal (Report No. 480/1/96). Water Research Commission.

[CR74] Viducich, J. M. G. (2015). Spillway staging and selective sediment deposition [Master’s thesis, Oregon State University]. Oregon State University Repository. Graduate Thesis Or Dissertation | Spillway Staging and Selective Sediment Deposition in Sand Storage Dams | ID: 1z40kx51c | ScholarsArchive@OSU (oregonstate.edu)

[CR75] WHO (2017). Guidelines for drinking-water quality: Fourth edition incorporating the first addendum.

[CR76] WHO & UNICEF (2017). Safely managed drinking water.

[CR77] WHO & UNICEF. (2022). Joint monitoring programme (JMP) for water supply and sanitation database. JMP. JMP (washdata.org)

[CR78] World Health Organisation (2016). Be smart drink water. A guide for school principals in restricting the sale and marketing of sugary drinks in and around schools.

[CR79] Wrisdale L, Michael Mokoena M, Sylvia Mudau L, Geere J-A (2017). Factors that impact on access to water and sanitation for older adults and people with disability in rural South Africa: An occupational justice perspective. Journal of Occupational Science.

