# Supplementary material for: Sand dam contributions to year-round water security monitored through telemetered handpump data
Source: Environ Monit Assess. 2023 Oct 17;195(11):1328. doi: 10.1007/s10661-023-11694-9 (PMC10582144; doi:10.1007/s10661-023-11694-9)
Supplement: Supplementary file 1 — (DOCX 180 kb) [file 10661_2023_11694_MOESM1_ESM.docx]

**Appendices**

Appendix A

| Sand Dam ID: Participant Number: Date: 1. What is your main source of water? And what is the purpose of use? |  |
| --- | --- |
| 2. Do you have access to the handpump? (i.e. is the handpump locked? Do you have the keys to access?) |  |
| 3. Do you have to pay to use the handpump from the sand dam? |  |
| 4. How far (distance) and how long does it take to walk to your closest handpump/water source? |  |
| 5. How many times a day do you travel to collect water from the water source, what time of day, and by what means? (i.e. bucket, trolley, etc.) |  |
| 6. Are there volume abstraction limitations from wells per household set by the organization/owner of the sand dams? |  |
| 7. Have you ever experienced the handpump running dry? (not enough water?) |  |
| 8. What does the water from the handpumps taste like versus scoop holes (if using)? |  |
| 9. Are there any suspected illnesses after using handpumps/scoop holes/other water sources relating to the sand dams? Do you treat the water? |  |
| 10. Are there any water vendors in the area abstracting water from the sand dams? |  |
| 11. Rainwater Harvesting tanks?  Other Comments: |  |

*Table S1: Community survey undertaken by Chan (2019) on sand dam handpumps and other water sources*

Appendix B

| **Pump** | **Daily litres range (L)** | **Daily rainfall range (mm)** | **Actual salinity (μs/cm)** | **Area of wall (m^2^)** | **Livestock use (%)** | **Abs limits** | **RWH tank (%)** | **Distance (km)** | **Perceived salinity (%)** | **Ever run dry** |
| --- | --- | --- | --- | --- | --- | --- | --- | --- | --- | --- |
| Athiani | 0-5348 | 0-66.145 | 2700 |  | 12.5 | No |  |  | 0.625 | No |
| Bodeni | 0-3863 | 0-63.394 | 545 | 79.04 | 50 | Yes | 18 | 0.8 | 0 | Yes |
| Ikanga | 0-1701 | 0-122.825 | 405 | 249.76 | 0 | No | 75 |  | 1 | No |
| Katelembu 16 | 0-3664 | 0-94.799 | 1155 | 156.24 | 0 | No | 33 | 0.5 | 0.666 | No |
| Katelembu 17 | 0-2597 | 0-28.362 | 1076 | 171.6 | 15 | No | 22 | 0.5 | 0.7 | No |
| Kee 13 | 0-10317 | 0-110.883 | 208 | 54.5 | 40 | Yes | 17 | 0.6 | 0 |  |
| Kee 14 | 0-5487 | 0-110.883 | 902 | 209.05 | 33 | No | 17 | 0.6 | 0 |  |
| Kinuvu | 0-9272 | 0-122.550 | 877 | 61.18 | 40 | Yes | 0 | 0.5 | 1 | No |
| Kinyenyoni | 0-2964 | 0-77.504 | 743 | 146.74 | 0 | Yes | 40 |  | 0 | No |
| Kithangaini 17 | 0-2820 | 0-117.058 | 1740 | 128.04 | 0 | No | 50 |  | 0 |  |
| Kithangaini 18 | 0-6271 | 0-102.368 | 391 | 420 | 0 | No | 50 |  | 0.333 |  |
| Kithunthi | 0-5492 | 0-126.019 | 1353 | 80.64 | 33 | Yes | 67 | 0.5 | 0.888 | No |
| Makutano | 0-5987 | 0-67.276 |  | 65 | 75 | Yes | 38 | 0.5 | 0.071 | Yes |
| Mapatano 17 | 0-7767 | 0-121.394 | 614 | 63.546 |  |  |  |  |  |  |
| Mapatano 18 | 0-3149 | 0-121.394 | 938 | 96.725 |  |  |  |  |  |  |
| Mbukilye | 0-2787 | 0-101.864 | 575 | 97.23 | 33 | Yes | 25 | 0.5 | 0 | No |
| Mukaso | 0-3565 | 0-110.883 | 872 | 66.36 | 60 | No | 40 |  | 0.5 | No |
| Mutula | 0-3871 | 0-87.971 | 1985 | 268.38 | 86 | Yes | 43 | 0.8 | 0.857 | No |
| Ndwae | 0-5466 | 0-119.905 | 1098 | 71.051 | 100 | No | 40 | 0.5 | 1 |  |
| Sindano | 0-3203 | 0-101.864 | 855 | 64.842 | 0 | Yes | 83 | 0.9 | 0 | No |
| Syakama 16 | 0-3136 | 0-60.886 | 1090 | 93.84 | 62.5 | Yes | 63 | 0.6 | 0.5 | Yes |
| Syakama 18 | 0-10906 | 0-60.886 | 1162 | 36.625 | 50 | Yes | 50 | 0.6 | 0.5 | No |
| Wendo | 0-4740 | 0-117.058 | 3150 | 43.51 | 0 | Yes | 33 |  | 1 |  |
| Wikwatyo | 0-20505 | 0-76.852 | 2690 | 72 | 100 | Yes | 20 | 2.1 | 1 | No |
| Woni 15 | 0-7833 | 0-64.835 | 427 | 139.95 | 0 | Yes | 46 | 0.5 | 0 | No |
| Woni 16 | 1-12473 | 0-61.038 | 1294 | 102.6 | 100 | Yes | 46 | 0.5 | 1 | No |

*Table S2: Raw variable values collected for each sand dam site used in the random effects models*
